# Zero Self-Harm app: a mobile phone application to reduce non-suicidal self-injury—study protocol for a randomized controlled trial

**DOI:** 10.1186/s13063-024-07932-1

**Published:** 2024-02-10

**Authors:** Evelyn Guerrero, Kate Andreasson, Lene Larsen, Niels Buus, Jette Louise Skovgaard Larsen, Jesper Krogh, Rasmus Thastum, Lone Lindberg, Katrine Lindblad, Annette Erlangsen, Merete Nordentoft

**Affiliations:** 1 Psychiatric Centre North Zealand, University Hospital of Hillerød, Dyrehavevej 48, 3400 Hillerød, Denmark; 2 Mental Health Centre Copenhagen, Gentofte hospitalsvej 15, 4th floor, 2900 Hellerup, Denmark; 3 Danish Research Institute for Suicide Prevention (DRISP), Mental Health Centre Copenhagen, Gentofte hospitalsvej 15, 4th floor, 2900 Hellerup, Denmark; 4 Department of Public Health, Aarhus University, Bartholins Allé 2, 8000 Aarhus C., Denmark; 5 Department of Mental Health, Johns Hopkins Bloomberg School of Public Health, Hampton House 624 N. Broadway 8th floor, Baltimore, MD 21205 USA; 6 Faculty of Medicine, Nursing, and Health Sciences, Monash University, Wellington Road, Clayton, 3800 Australia; 7 Center of Mental Health Research, Australian National University, Building 63, Canberra, ACT 2601 Australia; 8 Children’s Rights National Association, Trekronergade 26, 2500 Valby, Denmark; 9 Psykiatrisk Praksis, Arresødalsvej 79, 3300, Frederiksværk, Denmark; 10 Centre of Eating Disorders and Self-Harm (VIOSS), Krumtappen 2, 2500 Valby, Denmark

**Keywords:** Non-suicidal, Self-injury, Self-harm, Randomized controlled trial, Self-help, Mobile phone application, Safety plan

## Abstract

**Background:**

Non-suicidal self-injury (NSSI) is a growing healthcare problem. Individuals with NSSI have an increased risk of suicidality. Due to stigma, they may self-injure in secret, which means they might not seek help until events have escalated to include suicidal ideation or a mental disorder. Interventions delivered via mobile phone applications (apps) have been linked to reductions in self-injury. This protocol outlines a trial, which examines whether the Zero Self-Harm intervention, consisting of an app for people with NSSI, can reduce the number of NSSI episodes, suicide ideation, and depressive symptoms.

**Methods:**

The trial will be conducted as a 6-month 2-arm, parallel-group, multicentre, pragmatic, randomized clinical superiority trial. The intervention group will receive the app and instructions on how to use it, while the control group will be allocated to a waitlist and allowed to download the app after 6 months. After inclusion, participants will be asked to complete questionnaires at baseline, 3 months, and 6 months. The primary outcome is the number of NSSI episodes during the preceding month, as measured at the 6 months follow-up with the Deliberate Self-Harm Inventory. A total of 280 participants, 140 in each arm, will be included.

**Discussion:**

This trial will assess the effectiveness of the Zero Self-Harm intervention to reduce the number of NSSI episodes. If effective, the app will have the potential to support a large group of people with NSSI. Considering the stigma related to NSSI, the fact that the app may be used in private and anonymously might make it an appealing and acceptable option for support. The app was developed in collaboration with people with lived experiences related to current and/or previous NSSI. As a result of this, the app focuses on minimizing harm, rather than stopping NSSI. This might enhance its utilization.

**Trial registration:**

ClinicalTrials.gov NCT04463654. Registered on 7 June 2020.

**Supplementary Information:**

The online version contains supplementary material available at 10.1186/s13063-024-07932-1.

## Background

Non-suicidal self-injury (NSSI) is defined in the fifth version of the Statistical and Diagnostic Manual of Mental Disorders (DSM-5) as “the deliberate, self-inflicted destruction of body tissue without suicidal intent and for purposes not socially sanctioned, includes behaviours such as cutting, burning, biting and scratching skin” [1]. Though difficult to determine, the overall prevalence of NSSI among adolescents, young adults, and adults is estimated to be 17%, 13.4%, and 5.5%, respectively, reported in meta-analyses [2, 3]. NSSI is characterized by an early onset; in a US sample, about 27% of participants reported that self-injurious behaviour was initiated around the age of 15–16 years, while 38.6% of participants commenced at the age of 17–24 years [4].

People with NSSI have higher risks of dying, in particular by suicide, when compared to the general population [5–7]. NSSI is, furthermore, associated with a range of mental health problems, such as anxiety, depression, eating disorders, and drug and alcohol abuse [8–11]. The most common method of NSSI is cutting, while other behaviours include scratching, head banging, breaking bones, and burning oneself [12]. The majority of people use more than one method. Using a range of methods and several episodes of self-injury behaviour are considered markers of severe NSSI [13]. NSSI can be an addictive behaviour; approximately half of the adolescents with NSSI have had multiple episodes of self-injury [14, 15]. NSSI is often hidden due to stigma and shame [11, 16, 17].

Individuals with high levels of impulsivity can act rashly when experiencing negative emotions and, in this respect, NSSI may serve as a coping strategy and provide a form of immediate relief [18]. This supposition is supported by the fact that individuals with NSSI report greater difficulties with emotion regulation [19, 20] and have high levels of emotion dysregulation [21].

It is estimated that only 1 in 8 persons present at hospital or seek help in the public health sector after NSSI [14, 15]. People with NSSI might not be considered in need of specialized treatment, implying that, when left untreated, the behaviour might escalate to a point where the person becomes suicidal or develops a mental illness [5, 6].

In addition to being low-cost, easily delivered, and scalable, self-help mobile phone applications (apps) for people with NSSI have shown promising findings in randomized controlled trials, including moderate reductions in self-reported self-cutting and self-injurious episodes; symptoms of depression and anxiety; and improvements in coping with unpleasant emotions and thoughts [22]. Still, evaluations conducted in different settings are warranted to determine the true effectiveness of apps in reducing NSSI [23].

Findings from qualitative studies suggest that the option of receiving immediate support at home and in an anonymous format enhances the acceptability of the app [22–24]. Features, such as daily mood tracking, have been seen as a way to improve emotional self-awareness [25]. In addition, self-help apps may support individuals with different severity levels of NSSI [26, 27].

The goal of the Zero Self-Harm (ZSH) intervention is to motivate people with NSSI to reduce their self-injurious behaviour rather than to completely stop it, which for many might seem unrealistic and deter them from using the app. The ZSH app is developed from the MyPlan app, which was developed in 2012 and whose effect is currently being investigated for its ability to reduce suicide ideation [28, 29]. MyPlan has been reviewed by users and clinical staff with promising qualitative feedback [30, 31].

The objective of this trial is to investigate whether adults with NSSI who receive the ZSH app have fewer NSSI episodes at 6-month follow-up when compared to adults with NSSI who do not receive the ZSH app in a randomized clinical superiority trial. We hypothesize that the ZSH app can help reduce the number of NSSI episodes by enhancing coping skills to better manage impulsive urges. Further, working with the app might reduce emotional dysregulation and trait impulsivity, which are core features of NSSI, and thereby lower associated symptoms, such as suicide ideation and depression.

## Methods and design

**Fig. 1:**
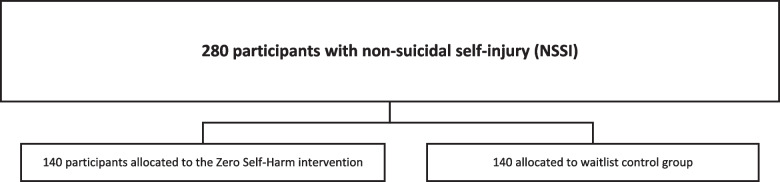
Flow diagram of the Zero Self-Harm intervention

The trial will be conducted as a 2-arm, parallel-group, multicentre, pragmatic, randomized clinical superiority trial. A total of 280 participants, 140 in each arm, will be included from clinical and non-clinical settings in Denmark and followed over 18 months (see Fig. 1). The intervention group will receive the ZSH app, while the control group will be allocated to a waitlist condition. The recruitment of participants was initiated in October 2020. See Table 1 for the entries to the World Health Organization Trial Registration Data Set.

**Table 1:** World Health Organization Trial Registration Data set

Data category	Information
Primary registry and trial identifying number	ClinicalTrials.gov NCT04463654
Date of registration in primary registry	07/07/2020
Secondary identifying number	Trygfonden 127390, The Regional Ethics Committee in the Capital Region of Denmark H 19024725
Source(s) of monetary or material support	TrygFonden reference number: 127390
Primary sponsor	Kate Aamund, Psychiatric Centre North Zealand; Denmark
Contact for public queries	KA: kateaamund@gmail.com
Contacts for scientific queries	KA: kateaamund@gmail.com
Public title	Zero Self-Harm - a Mobile Phone Application to Reduce Non-suicidal Self-injury
Scientific title	Zero Self-Harm - a Mobile Phone Application to Reduce Non-suicidal Self-injury: A Randomized Clinical Superiority Trial
Country of recruitment	Denmark
Health condition(s) or problem(s) studied	Self-injurious behavior
Intervention(s)	Device: Zero Self-Harm app, safety plan app for smartphones
Key inclusion and exclusion criteria	Ages eligible for study: ≥ 18 years; sexes eligible for study: both Inclusion Criteria: Engagement in two or more episodes of NSSI in the past month, have a smartphone (IPhone or Android phone), fluent in Danish, skills sufficient to enable use of the Zero Self-Harm app, provide an informed written consent. Exclusion Criteria: Lack of informed consent, further planned specialized treatment focused on NSSI.
Study type	Allocation: Randomized Interventional Model: Parallel Assignment Masking: Single (Outcomes Assessor) Primary Purpose: Prevention
Date of first enrolment	October 15, 2020
Target sample size	280
Recruitment status	Not recruiting
Primary outcome(s)	Number of self-harm episodes: Deliberate Self Harm Inventory [Time Frame: 6 months]
Key secondary outcome(s)	Suicide ideation: Becks Suicide Ideation Scale [Time Frame: 6 months] Depression: Major Depression Inventory (MDI) [Time Frame: 6 months] Well-being: WHO Well-being Index [Time Frame: 6 months] Self-esteem: Rosenberg's Self-Esteem scale [Time Frame: 6 months]

### Recruitment

We will strive to include individuals with a wide range of NSSI, from those with shorter duration and milder symptoms who might not be in contact with mental healthcare facilities to those with more severe and chronic NSSI who are already attending treatment at Danish mental health facilities. Non-profit organizations, such as Danish Mental Health Fund (Psykiatrifonden), The Danish National Association for Eating Disorders and Self-harm (Foreningen Spiseforstyrrelser og selvskade), and the Danish telephone helpline for people with suicide thoughts (Livslinien), as well as municipal service centres, educational institutions for individuals aged ≥18 years, psychiatric inpatient units and outpatient clinics, and psychiatric and general emergency departments will be contacted with regard to the referral of potential participants. Furthermore, the trial will be announced through non-profit organizations’ websites and social media platforms and presented at educational institutions for individuals ≥ 18 years old. The research assistant responsible for recruitment will routinely visit relevant locations, such as in-patient clinics in the Capital Region of Denmark, and give reminders to participating venues through e-mails and phone calls.

### Criteria for inclusion and exclusion

Individuals who fulfil the following criteria are included: (1) have had two or more physical NSSI episodes within the past month, (2) own a smartphone (iPhone or Android), (3) are fluent in Danish, (4) are able to install and use the ZSH app, (5) provide informed written consent, and (6) are ≥18 years old. Individuals who (1) are attending specialized treatment for NSSI (Dialectical Behavioral Therapy), e.g. at newly established outpatient clinics in the Capital Region of Denmark, or (2) are currently being involuntarily treated will not be enrolled.

### Enrollment and randomization

Interested individuals will be encouraged to contact the research assistant through phone or e-mail. They will be provided with verbal and written information about the study, in which it is emphasized that participation is voluntary and that they may withdraw their consent at any time. They may take time to consider or discuss the participation with a next of kin before deciding whether to enrol in the study. During the enrollment phase, the potential participants will be interviewed by using the Mini-International Neuropsychiatric Interview (MINI) [32] and informed consent will be collected via an e-mail from REDCap [33] to the secure digital platform e-Boks where Danish citizens receive verified information [34]. The research assistant will make sure to get the informed consent in the enrollment phase. The participant information materials and informed consent form are available from the corresponding author.

After enrollment, participants will be administered the baseline questionnaires. Subsequently, they will be randomly assigned to either the intervention or the control group in a 1:1 allocation, using REDCap’s feature for a computer-generated sequence randomization. The randomization will be stratified by sex and number of NSSI episodes, as measured at baseline by the Deliberate Self Harm Inventory, to avoid overrepresentation of participants with a high number of NSSI episodes in one group. This stratification will be also facilitated through REDCap. The research assistant will generate the allocation sequence in REDCap after enrolling the participant and inform the participant which group, they are assigned to.

As part of the enrollment process, all participants will be informed that they can contact the research assistant in case of questions or technical problems. Participants will also receive information on where they can seek help if needed, for instance through helplines and the emergency services, such as 1318 and 112.

### Intervention

Participants allocated to the intervention group will receive the ZSH app. The app was adapted to the target group through focus-group interviews with people with lived experiences related to NSSI. Based on their suggestions, the app consists of following elements: (1) a tool for listing warning signs of self-injurious thoughts; (2) a tool to identifying coping strategies when self-injurious thoughts arise, including a list of suggestions formed by users; (3) a daily mood tracking option, including pop-up reminders; (4) a place to write down dreams and how to realize them; (5) a place for collecting pictures, music, YouTube videos, which can cheer one up, and write positive message for oneself to practise a positive self-image; (6) a place to reflect on previous NSSI-episodes and read stories from other users; (7) direct phone links to emergency services and nearest map directions for emergency departments. The app is listed under an undisclosed name in Google Play and Apple Store to avoid that people not included in the project will find the ZSH app and start using it.

As part of active onboarding measures to the ZSH app, participants will receive an e-mail instructing them how to download the ZSH app from Google Play or Apple Store. Once an account has been set up, a pop-up message will invite the participant to watch instruction videos explaining to them how to use the app. Participants in the intervention group are also invited to a phone call 1 week after inclusion by the research assistant for providing further information on how to use the ZSH app and to encourage the participants to use the app. Throughout the trial, the research assistant will be available to answer questions regarding the ZSH app and give technical support.

Participants allocated to the waitlist control group will be invited to download the ZSH app once answers to the last questionnaire have been submitted after 6 months.

### Outcomes

**Fig. 2:**
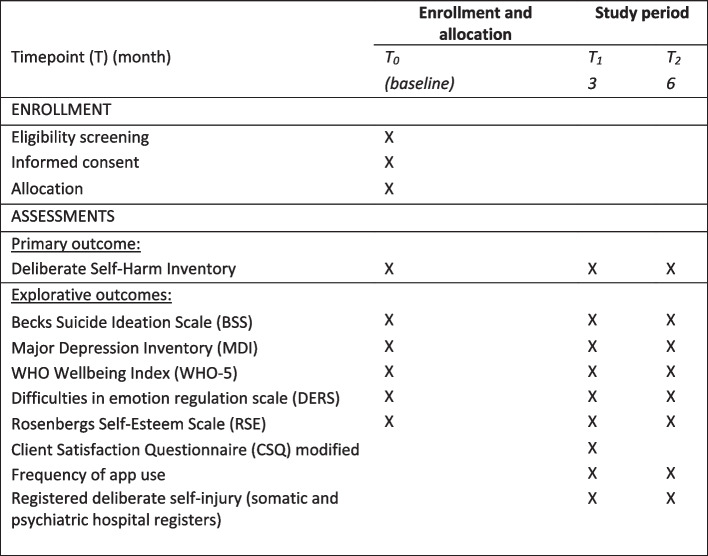
SPIRIT overview of enrollment and data collection

The primary outcome, the number of NSSI episodes during the preceding month, will be measured using the validated 17-item Deliberate Self Harm Inventory (DSHI) [35] at the 6-month follow-up (see Fig. 2). This measure includes questions on a number of NSSI episodes for 17 frequently used methods, e.g. cutting, burning, and scratching. As the original inventory uses a recall period of 4 months, a modified version adapted to 1 month will be used. This modification was done to limit any recall bias regarding the number of NSSI episodes [36]. The DSHI questionnaire will be administered at baseline and 3- and 6-month follow-up.

Explorative outcomes include suicide ideation, depressive symptoms, quality of life, and self-esteem. Suicide ideation will be measured by the Becks Suicide Ideation Scale (BSS), a 21-item self-report questionnaire measuring suicidal ideation [37]. Depressive symptoms will be measured using the Major Depression Inventory (MDI) [38], a short questionnaire consisting of 12 items capturing depressive symptoms. It can be scored as a diagnostic tool, but also used as a measure of severity as the sum of the item scores [38]. Quality of life will be measured by the WHO Wellbeing Index (WHO-5), a widely used brief questionnaire to measure subjective psychological wellbeing, consisting of 5 questions [39, 40]. Self-esteem will be measured by Rosenberg’s Self-Esteem Scale (RSE), which consists of 10 items measured on a Likert scale [41]. All explorative outcomes will be administered at baseline and 3- and 6-month follow-up.

User satisfaction will be measured by a modified Client Satisfaction Questionnaire (CSQ-8) at 3-month follow-up among participants allocated to the intervention group. An overview of the assessments and timepoints in the trial is provided in the SPIRIT figure (see Fig. 2). See Additional file 1 for the SPIRIT checklist [42].

### Safety measure

To assess potential harmful effects, the 20 first participants who receive the ZSH app will be contacted after completing the 3-month questionnaire for an interview regarding their experiences. The interviewed participants will be asked systematically if they had any negative experiences using the app. If any adverse effect (AEs) or serious adverse effects (SAEs) in relation to the use of the self-help app occurs, it will be reported to the Trial Steering Committee and it will be decided which action will be made, either minor changes to prevent other future events or to stop the trial.

### Data collection

The participants will be administered questionnaires at baseline (*T*_0_) and at two follow-up time point (*T*_1_=3 months and *T*_2_= 6 months). At baseline, the participants will undergo the MINI interview and there will be socio-demographic questions, including age, gender, and use of psychiatric services. The MINI is administered by the research assistant. All other questions will be self-administered and sent to the participant by e-mail at baseline and follow-up. All the participants will be allocated an individual trial identification number and all data will be collected in REDCap in a central computer storage under Mental Health Services in the Capital Region of Denmark.

To promote participant retention, robot-generated reminders will be sent out 1 week after the dissemination of questionnaires. If participants have not completed the questionnaire a week after the reminder was sent out, the research assistant will contact the participant and encourage them to answer the questionnaires and assist with any technical problems. All the participants will receive a minor gift card to a coffee shop once the questionnaire is completed. If a participant wishes to discontinue their participation, their data will be deleted. Still, the number of discontinuations will be reported in the Consort flow diagram. The Project Management Group is planned to meet on a weekly basis and the Trial Steering Committee will meet approximately every third month to review trial conduct and progress. The Ethical Committee and the independent Data Monitoring can review trial conduct throughout the whole trial period.

To secure data confidentiality, data on participants will be stored in a logged and closed computer drive in the Mental Health Services in Capital Region of Denmark. It will be stored until 5 years after the trial has finished. Afterwards, data will be permanently deleted.

### Sample size

Based on previous estimates, a reduction of 3.5 NSSI episodes is expected in the intervention group after the 6-month follow-up compared to participants in the waitlist control group at follow-up after 6 months [28, 43, 44]. A post-intervention standard deviation of 9 is assumed based on previous findings [43, 44]. If the true difference between the intervention and control groups is 3.5, a total of 140 participants should be included in each group in order to reject the null hypothesis with a power of 90% and a type I error probability of 0.05, implying a total of 280 participants.

### Statistical analysis

The analysis will be conducted according to the intention-to-treat principle. All participants will be included in the analysis according to group assignment, regardless of adherence. For the analysis of the primary outcome, the number of NSSI episodes during the preceding month, as measured at the 6-month follow-up, we will assess the intervention effect using linear regression with group allocation as the independent variable. If data from more than 5% of the participants are missing at 6 months, we will use multiple imputation to account for missing data. Imputation will be based on baseline NSSI, gender, age, and baseline variables associated with missingness. Secondary analysis will include baseline NSSI, age, gender, and baseline characteristics which differ between groups at baseline. Confidence intervals will be presented as well as significance level. All tests will be two-tailed and *p* values below 0.5 will be considered significant and interpreted with respect to the hierarchy of hypothesis recognizing that all outcomes, apart from the primary, are exploratory.

The analysis will be conducted using SPSS, version 22.0.

### Blinding

Due to the nature of the intervention, neither the participants nor the research assistant can be blinded to the intervention. Additionally, the collection of the primary and explorative outcomes will not be blinded. At study completion, a researcher not involved in the research project will extract data from REDCap into two separate files where group allocation will be coded as A and B to ensure blinding during the period of data analyses, interpretation, and drafting of the first manuscript. The blinding will be unmasked once the conclusion has been written.

## Discussion

Emerging research suggests that specialized apps targeting self-injurious behaviour, which can be used anonymously and in private, have the potential to help people with NSSI [23, 24]. However, randomized controlled trials confirming the effectiveness of these apps in different settings are warranted [23, 24].

The ZSH intervention has several strengths. First, by involving stakeholders and people with lived experiences in the development of the app, the app’s content is likely to be relevant to the target group. Second, by having a focus on minimizing harm, the app might be more appealing and acceptable to a broader group of users. Third, by listing the app under an undisclosed name, contamination of the trial’s data will be limited.

Potential limitations should be noted. Firstly, although the app was developed in collaboration with individuals with lived experiences, it may not address the needs of everyone engaging in NSSI. Individuals with NSSI are characterized by broad age spans, diagnoses, severity and versatility of NSSI, and co-morbidities. Although it might be beneficial to also collect feedback and insights from next of kin and professionals, this was beyond the scope of this intervention.

Secondly, it is important to acknowledge that the use of the app might maintain or possibly worsen NSSI. To investigate this potential limitation, the first 20 participants will be asked after 3 months about whether they have had any negative experiences or side effects of the app. Other potential limitations could be linked to how users learn how to use the app, which for some participants can be difficult and overwhelming. It might result in some participants using the app only a few times or not at all. Even though a thorough onboarding process is carried out, more continuous and structured support might improve the usage of the app. Another way of combining a more structured support with technology could also have been by using for instance automatically collected smartphone sensor data, which is not included in this trial. In general, having a self-help app as an intervention can be challenging since technological development is an on-going process. Some of the app features designed to be user-friendly can potentially become outdated during the trial. Once the trial is started, the app cannot undergo significant changes, and this could limit the possibility of continuously ensuring the relevance of the app and its design.

Thirdly, the broad recruitment strategy might imply that participants using a range of mental health services will be included, which potentially could affect the treatment outcome. Persistent self-harm behaviours and more severe underlying psychopathology could affect the effect of the app. By collecting information on the received treatment, we can adjust for this in the analysis. However, there might be a difference in how the general experience of the app is, depending on the knowledge level of NSSI and the degree and duration of the NSSI. The broad variation of participants, which includes both individuals with milder symptoms of NSSI and more severe and chronic NSSI, can make it difficult to distinguish a potential difference in maybe the usage of the app, how relevant the app is perceived and the outcome of the app. Also, specialized treatment for NSSI is an exclusion criterion which can furthermore make the recruitment more difficult.

The questionnaires in the trial cover different topics such as NSSI, suicidal ideation, depressive symptoms, and self-esteem which for some of the participants can be difficult to reflect on and quantify. Avoidance of these topics might occur and make it more difficult to get the questionnaires answered, especially since it is self-administered questionnaires. The questionnaires are sent out three times with a 3-month interval and that can also be an obstacle since it requires time and effort which can lead to participant dropout.

Finally, neither the participants nor the research assistant will be blinded to the assignment of treatment condition due to the nature of the intervention. However, the research assistant will remain blinded during the analysis and drafting of the manuscript.

A worsening of the condition of a participant may occur during the trial. To ensure the participants know where to seek help, the research assistant will inform all participants in the enrollment process about relevant support options. As part of the onboarding process, the intervention group will be offered an introduction by phone on how the app can be used as a safety plan and important phone numbers to emergency services are pre-installed in the app, etc. The research assistant will be available to answer questions regarding the app or the questionnaires and give technical support throughout the trial.

### Trial status

The trial began to recruit participants in October 2020 and the recruitment and enrollment have continued until September 2023. The last questionnaire (the 6-month follow-up) will be sent to the last participant on 22 February 2024. Due to delays caused by the COVID-19 pandemic, change of research staff members and maternity leave, it has not been possible to submit this study protocol earlier. The protocol version number is 1.2, dated 30 July 2020.

### The Trial Steering Committee

The Trial Steering Committee consists of Principal Investigator Kate Andreasson Aamund, Professor Merete Nordentoft, Professor Niels Buus, Associate professor Annette Erlangsen, Jesper Krogh, Jette Skovgaard Larsen, and research assistant Evelyn Guerrero, and in close collaboration with the head of Psychiatric Centre North Zealand Chief of Centre Henrik Søltoft. The Trial Steering Committee will hold a regular meeting every third month, and in case of urgency, there will be convened another meeting. If protocol modifications are required, such as changes to eligibility criteria, outcomes, and analyses, the Trial Steering Committee will be notified, and an agreement secured. Any changes will also be updated in clinicaltrials.gov.

### Supplementary Information

**Additional file 1.** SPIRIT checklist.

## Data Availability

Following the Consort guidelines, we will publish positive, neutral, and negatives findings. The dissemination plan for the trial is regardless of results it will sought to be published in scientific journals and we will participate in national and internationals congresses. Any data required to support the protocol can be supplied on request. The dataset is available from the corresponding author on reasonable request.

## Abbreviations

NSSI: Non-suicidal self-injury
app: Mobile phone application
ZSH: Zero self-harm
MINI: Mini-International Neuropsychiatric Interview
